# Postpartum prostaglandin F2**α** administration affects colostrum yield, immunoglobulin G, and piglet performance

**DOI:** 10.5713/ajas.20.0187

**Published:** 2020-10-13

**Authors:** Photcharaphan Maneetong, Chutikan Srisang, Naritsara Sunanta, Praeploy Muchalintamolee, Pachara Pearodwong, Junpen Suwimonteerabutr, Fabio De Rensis, Padet Tummaruk

**Affiliations:** 1 Department of Obstetrics, Gynaecology and Reproduction, Faculty of Veterinary Science, Chulalongkorn University, Bangkok 10330, Thailand; 2 School of Agricultural Resources, Chulalongkorn University, Bangkok 10330, Thailand; 3 Swine Reproduction Research Unit, Chulalongkorn University, Bangkok 10330, Thailand; 4 Department of Veterinary Medical Science, University of Parma, Parma 43121, Italy

**Keywords:** Colostrum, Immunoglobulin, Pig, Postpartum, Prostaglandin

## Abstract

**Objective:**

Current study was conducted to determine the effect of postpartum prostaglandin F2α (PGF2α) administration on colostrum and milk yield, colostrum immunoglobulin G (IgG) and piglet growth performance.

**Methods:**

In total, 36 sows were included in the experiment. The sows were classified into two groups: i) control (n = 11) and ii) PGF2α (n = 25). Sows in the PGF2α group received 10 mg of PGF2α within an hour after farrowing. The body weight of piglets was measured at 0 and 24 h after birth to estimate colostrum consumption. Colostrum was collected at 1 and 24 h after farrowing to determine IgG concentrations. For milk yield study, the remaining sows in the PGF2α group (n = 23) were divided into two subgroups: i) single PGF2α (n = 12) and ii) multiple PGF2α (n = 11). In the multiple PGF2α, the sows received repeated doses of PGF2α at seven and 14 days postpartum. The piglets’ body weight was measured at 0, 1, 5, and 20 days of age. The milk yield of the sows was calculated.

**Results:**

Colostrum yield of sows averaged 5.62±2.25 kg. Sows treated with PGF2α postpartum had a higher colostrum yield than control (7.01 and 5.12 kg, p<0.05). The concentration of IgG in colostrum at 24 h in the PGF2α group was higher than the control (31.6 and 17.4 g/L, p<0.05). For primiparous sows, milk yield was highest in the sows treated with multiple doses of PGF2α during lactation and lowest in control sows (10.25 and 7.61 kg, p<0.05). Colostrum intake was higher in the treatment than the control groups (+56.7 g, p<0.05). Primiparous sows treated with multiple doses of PGF2α had a higher litter weight than controls (p<0.01).

**Conclusion:**

Postpartum treatment with PGF2α improved colostrum yield and IgG in multiparous sows and increased colostrum intake of piglets. Multiple administration of PGF2α improved the milk yield and increased litter weight of piglets in primiparous sows.

## INTRODUCTION

Inadequate colostrum consumption of piglets is one of the most important underlining causes of piglet preweaning mortality [1]. A previous study indicated that at least one-third of sows do not produce enough colostrum to fulfil the needs of their offspring [2]. Therefore, various management tools to increase colostrum consumption of piglets need to be investigated. The ways that have been suggested to increase piglet colostrum consumption include: i) increase the ability of piglets to suckle; ii) reduce within-litter variation in birth weight; and iii) increase the quantity of colostrum that sows produce [2]. However, during the past decade, an increase in litter size in modern hyper-prolific genetic sows led to a decrease in body weight at birth of the piglets [3,4]. Piglets with low birth weight had a reduced capacity to suckle and compete for a teat [5]. Therefore, care of newborn piglets and postpartum sows to ensure that all the piglets receive adequate colostrum intake is of utmost importance.

Prostaglandin F2α (PGF2α) is an important substance regulating the function of the corpora lutea in female pigs [6]. In practice, PGF2α is commonly used for farrowing induction in order to facilitate cross fostering management [7,8]. However, PGF2α also stimulates myometrial contraction [6]. Thus, administration of PGF2α in postpartum sows can eliminate residual products and infectious debris that occur during farrowing. Vanderhaeghe et al [9] found that administration of PGF2α in postpartum sows increased piglet body weight at weaning and increased litter size at subsequent farrowing. To our knowledge, the mechanism of how the use of PGF2α in postpartum sows is associated with the reproductive performance of lactating sows has not been completely elucidated. Moreover, multiple doses of PGF2α have been used to treat purulent vaginal discharge in postpartum dairy cows [10]. However, the results concerning the efficacy of PGF2α treatment in cows are still controversial for a number of reasons, e.g. different case definition, low number of animals per group and lack of negative control [11]. In sows, the influence of multiple treatments of PGF2α during lactation on milk production and piglet performance have not been investigated. The present study aimed to determine the effect of PGF2α administration postpartum on colostrum production of sows, colostrum intake of piglets and immunoglobulin G (IgG) concentration in the sow colostrum and to determine the influence of multiple treatments of PGF2α during lactation in sows on their milk yield and piglet performance until weaning.

## MATERIALS AND METHODS

### Animal care

This experiment followed the guidelines of The Ethical Principles and Guidelines for the Use of Animals for Scientific Purposes by the National Research Council of Thailand and was approved by the Institutional Animal Care and Use Committee in accordance with the university regulations and policies governing the care and use of experimental animals (animal use protocol no. 1831057).

### Experimental design and herd

This study was carried out in a swine breeding herd with 1,500 sows, located in the eastern part of Thailand between June and July 2018. The average daily minimum to maximum temperatures during the experimental period was 24.7°C to 32.9°C with an average humidity of 83.8%±4.2% (range 76.0% to 95.0%). The breed of sows was crossbred Landrace×Yorkshire F1 females with parities between one and seven. Sows were kept in a conventional open-housing system. The sows were transferred to the farrowing house 5 to 7 days before the estimated farrowing date. The sows were individually housed in crates until weaning. Weaning occurred at 22.8±2.3 days after farrowing. In total, 36 Landrace×Yorkshire crossbred sows were included in the experiment (13 primiparous and 23 multiparous sows). The sows were classified into two groups: i) control (n = 11) and ii) PGF2α (n = 25). Sows in the PGF2α group received 10 mg (2 mL) of PGF2α intramuscularly (5 mg/mL Dinoprost, Enzaprost Vet, CEVA Animal Health, Libourne, France) within an hour after the end of farrowing (day 0). The body weight of piglets was measured immediately after birth and at 24 h after birth to estimate colostrum consumption of each piglet. Colostrum was collected at 1 h and 24 h after farrowing. IgG concentrations in colostrum were determined using enzyme-linked immunosorbent assay (ELISA). For the milk yield study, the remaining sows in the PGF2α treated group (n = 23) were divided into two subgroups: i) single PGF2α (n = 12) and ii) multiple PGF2α (n = 11). In both subgroups, the sows received PGF2α at day 0, but the sows in the multiple PGF2α subgroup received PGF2α treatment again at days 7 and 14 postpartum. The piglets were identified by using an ear tattoo and their individual body weight was determined at 0, 1, 5, and 20 days of age. The milk yield of the sows was calculated by using a Bayesian hierarchical model [12]. Due to some exclusion criteria (i.e., live-born <8, n = 6, and received farrowing assistances, n = 4), the analyses data on colostrum yield of sows was based on 26 normal farrowing sows (n = 10) and PGF2α (n = 16) treatment.

### Supervision of parturition process and postpartum management

Parturition was carefully monitored for 24 h per day by the research team. Briefly, the sows were disturbed as little as possible during parturition. Birth assistance was performed only when an interval of 45 to 60 min had elapsed after the birth of the previous piglet. Birth assistance included the stimulation of uterine contractions by intramuscular administration of 20 IU oxytocin and when required, manual extraction of the fetus (n = 2). Birth assistance was required for one sow in the control group, two sows in the single PGF2α group and one sow in the multiple PGF2α group. Sows that required birth assistance and used oxytocin were excluded from the analysis concerning colostrum yield (n = 4). At farrowing, the date of farrowing, the total number of piglets born per litter (TB), number of piglets born alive per litter (BA), number of stillborn per litter, and number of mummified foetuses per litter were recorded. The duration of parturition of individual sows was also recorded. Parturition duration was defined as the period from the first to the last piglet delivery. After the end of the farrowing process, all sows were treated with a non-steroidal anti-inflammatory drug (Tolfédine, Vetoquinol S.A., Lure Cedex, France) for two days postpartum, an antibiotic (amoxicillin 15 mg/kg intra-muscularly, Amoxilin LA, Univet Co. Ltd., Cavan, Ireland) at days 1, 3, and 5 postpartum and vitamins (217.8 mg/sow intramuscularly using Fercobsang, Vetoquinol S.A., Lure Cedex, France) on day 2 postpartum. The routine procedures performed on piglets included weighing, teeth clipping and the intramuscular administration of 200 mg of iron dextran (ABI-DEX 100, Abic Ltd., Ramat Gan, Israel) on the first day of life. On the second day of life, 25 mg of toltrazuril was administered orally (Pharmazuril 5%, Pharmatech Co., Ltd., Ratchaburi, Thailand). Health management of sows was supervised by veterinarians. After farrowing, the sows were vaccinated against porcine parvovirus (Porcilis PARVO, MSD Animal Health, Kenilworth, NJ, USA). Whole-herd vaccinations against foot and mouth disease, classical swine fever (CSF, Pestiffa, Merial Asia Pte Ltd., Central Area, Singapore), Aujeszky’s disease (Novoyesky, Syva Laboratorios, Leon, Spain) and porcine reproductive and respiratory syndrome (PRRS) (Prime Pac PRRS RR, Merck Animal Health, Kenilworth, NJ, USA) were performed in all gilts and sows every four months. The piglets were vaccinated against PRRS, given a combined vaccine of Mycoplasma and PCV2 (Porcilis PCV M Hyo, MSD Animal Health, USA) and CSF at two, three and five weeks of age, respectively.

### Progesterone determination

Blood samples were collected from the jugular vein at days 0, 7, and 21 after farrowing and kept at −20°C before progesterone assay. An enzyme-linked immunosorbent assay (ARBOR assays Inc., Ann Arbor, MI, USA) was used to determine the progesterone concentrations. All the samples were analysed in duplicates. Briefly, a solid phase high-binding clear microplate was coated with goat anti-mouse IgG 0.01 g/L. The standard progesterone hormone (12.72 nmol/L) was added to the well. Two-fold dilution was performed to obtain standard progesterone concentrations of 200.0, 100.0, 50.0, 25.0, 12.5, 6.25, 3.12, 1.56, and 0.78 pg/well. A 50 μL of standard and samples (dilute 1:30) were pipetted into the microplate, in duplicate. Progesterone-HRP (25 μL) was added in all tubes. Progesterone antibody (25 μL) was added in all tubes except the blank well. The plate was covered with a plastic sheet and shaken for 2 h at room temperature (25°C). The plate was washed five times before TMB peroxidase substrate (3,3′,5,5′-tetramethylbenzidine, 100 μL) was added. After 20 min, a 50 μL stop solution (1 N HCl) was added. Optical density (OD) was determined by using an ELISA reader at 450 nm. The OD was transformed to a percentage of binding by comparing with a standard curve. The concentration of progesterone (ng/mL) = ([progesterone (pg/well) ×dilution factor]×20)/1,000. The sensitivity of the assay was 0.19 nmol/L. The inter- and intra-assay coefficients of variation were 3.47% and 2.78%, respectively.

### Colostrum and immunoglobulin G determination

The colostrum samples were manually collected from all active teats at 1 h and 24 h after the onset of farrowing. For IgG determination, the colostrum samples were pooled in tubes of 30 mL, stored in a Styrofoam box with an ice pack (4°C) and then cryopreserved at −20°C. The IgG concentration in the sow colostrum was determined according to our previous study [13]. The concentration of IgG was presented in g/L. The inter- and intra-assay coefficients of variation were 6.9% and 2.3%, respectively.

### Determination of colostrum consumption and colostrum yield

Body weight of the newborn piglets was measured immediately after birth, using a digital scale (SDS IDS701-CSERIES, SDS (Yangzhou) Digital Scale Co. Ltd., Yangzhou, China). The newborn piglets were identified by an ear tattoo. Colostrum intake of each individual piglet was estimated by an equation published by Theil et al [14]: Colostrum consumption (g) = −106+2.26 WG+200 BW_B_+0.111 D–1414 WG/D +0.0182 WG/BW_B_, where WG is piglet weight gain over 24 h (g), BW_B_ is birth weight (kg) and D is the duration of colostrum suckling (min). The colostrum yield of the sows was defined as the sum of individual colostrum consumption by all piglets in the litter.

### Piglet body weight, average daily weight gain, litter weight gain and estimation of milk yield

Individual piglet’s body weight was determined at birth (i.e. before colostrum ingestion) (day 0) and at days 1, 5, and 20. The piglets body weight at weaning and litter weight at weaning were collected. Average daily weight gain (ADG, g/d) of the piglets from five days to weaning was calculated as: ADG (g/d) = (body weight at weaning – body weight birth/lactation length)×1,000. Individual milk yield of the sows was estimated by an equation published by Hansen et al [12]: milk yield (kg) = 2.23+0.05 (litter size – 9.5)+0.23×(average litter daily weight gain [kg/d]) – 2.5.

### Statistical analyses

Statistical analyses were performed by using SAS (SAS version 9.4, Cary, NC, USA) [15]. Descriptive statistics on sow data (n = 36) and piglet data (n = 407) were analysed by using the MEANS procedure of SAS. For continuous variables, the descriptive data were presented as means±standard deviation (SD) and range of the variables. The analysed data were presented as least square means±standard error of the means (SEM). Frequency distribution of categorical traits including the IgG concentration and colostrum intake of piglet classes was conducted by using the FREQ procedure of SAS. The difference between IgG at 1 and 24 h postpartum was compared within sow by using a paired *t* test. Additionally, the declining of IgG concentrations during the first 24 h postpartum in control and treatment groups for either primiparous or multiparous sows was analysed by using paired *t* test.

The sow traits determined within 24 h after farrowing including farrowing duration (n = 36), expulsion interval (i.e. farrowing duration/TB, n = 36), colostrum yield (n = 29), IgG at 1 h (n = 34) and 24 h (n = 23), TB (n = 36), BA (n = 36) and serum progesterone at day 0 were analysed by using the general linear model (GLM) procedure of SAS. Factors included in the statistical models consisted of treatment groups (control and PGF2α), parity (primiparous and multiparous sows) and their interactions. For farrowing duration, the statistical model was also included the total number of piglets born per litter in the model as a co-variance. Least square means were obtained from each class of the factors and were compared by using least significant difference tests. Additionally, an adjusted colostrum yield was re-analysed by excluding the litters having less than eight piglets (n = 6) from the statistical model. The remaining data on sow colostrum (n = 26) was re-analysed using the same procedure as describe above.

The piglet traits determined within 24 h after birth including birth weight (n = 406) and colostrum intake (n = 325) were analysed by using the GLM procedure of SAS. Factors included in the statistical models consisted of treatment (control and PGF2α groups), parity (primiparous and multiparous sows) and their interactions. For colostrum intake, birth weight of the piglets was also included in the statistical model as regression. Least square means were compared by using least significant difference tests.

The sow traits determined at weaning including milk yield (n = 33), litter weight at 20 days of lactation (n = 36), litter weight gain (n = 35), body weight of piglet at weaning (n = 36), ADG (n = 35) and serum progesterone at seven (n = 35) and 21 days (n = 32) postpartum were analysed by using multiple analysis of variance under the GLM procedure of SAS. Factors included in the statistical models consisted of treatment (control, single PGF2α and multiple PGF2α), parity (primiparous and multiparous sows) and their interactions. Least square means were obtained from each class of the factors and were compared by using least significant difference tests. For all analyses, p<0.05 was regarded as being statistically significant.

## RESULTS

### Reproductive performance

On average, TB and BA were 12.4 and 11.1 piglets/L, respectively. The farrowing duration of sows averaged 201.7±108.0 min (mean±SD). The duration of farrowing did not differ between control and PGF2α groups (151.5±26.9 and 196.6± 17.9 min, respectively, p = 0.173). However, after the exclusion of the litters having less than eight piglets born alive from the analyses (n = 6), the adjusted farrowing duration of sows was 220.3±102.6 min (range 61.8 to 421.8 min). The adjusted duration of farrowing did not differ between control and PGF2α groups (197.1±48.8 and 199.4±20.2 min, respectively, p = 0.965). Similarly, the expulsion interval did not differ between control and PGF2α groups (13.3±2.4 and 15.6±1.6 min, respectively, p = 0.173). However, multiparous sows had a longer farrowing duration than primiparous sows (245.7±17.5 and 124.4±24.7 min, respectively, p<0.001). The expulsion interval was also longer in multiparous sows than primiparous sows (20.4±1.7 and 8.4±2.3 min, respectively, p<0.001).

### Colostrum yield and colostrum intake

The first 24 h colostrum yield of sows nursing 10.7 piglets per litter was 5.62±2.25 kg and the colostrum intake of each piglet was 601.8±152.1 g (mean±SD). Sows treated with PGF2α postpartum had a higher colostrum yield than control sows (6.41±0.45 and 3.78±0.57 kg, respectively, p<0.001). Colostrum yield of multiparous sows was higher than primiparous sows (5.90±0.45 and 4.28±0.57 kg, respectively, p<0.01). On average, body weight at birth of the piglets did not differ significantly between control and treatment groups (1.38 and 1.33 kg, respectively, p>0.05). The colostrum yield of sows after PGF2α treatment compared with control sows by parity groups is presented in Table 1. For all litters, primiparous sows treated with PGF2α postpartum yielded a higher amount of colostrum than control sows (6.35±0.68 and 2.22±0.91, respectively, p<0.001). However, after the exclusion of the litters having less than eight piglets born alive from the analyses (n = 6), the adjusted colostrum yield of sows was re-analysed. The adjusted colostrum yield was 6.65 ±1.71 kg (range 2.86 to 9.11 kg). Similarly, sows treated with PGF2α postpartum still had a higher colostrum yield than control sows (Table 1). Moreover, multiparous sows treated with PGF2α postpartum had a significantly higher colostrum yield than control multiparous sows (+1.89 kg, p<0.05, Table 1). Likewise, a similar tendency was also observed in primiparous sows (+1.91 kg) although the different was not significant (p>0.05). However, the adjusted colostrum yield did not differ significantly between primiparous and multiparous sows (5.65±0.84 and 6.48±0.36 kg, respectively, p> 0.05).

Colostrum intake of piglets varied from 66.3 to 987.9 g. Of all the piglets, 8.0% (n = 26) had a colostrum intake less than 400 g. Factors influencing colostrum intake of the piglets included PGF2α (p = 0.013) and the body weight at birth of the piglets (p<0.001). Colostrum intake by piglets was higher in the treatment than the control groups (+56.7 g, p = 0.013) (Table 1). Colostrum intake of the piglets increased on average 17.5 g (p<0.001) with each increase of 100 g in birth weight.

### Immunoglobulin G concentration

**Figure 1:**
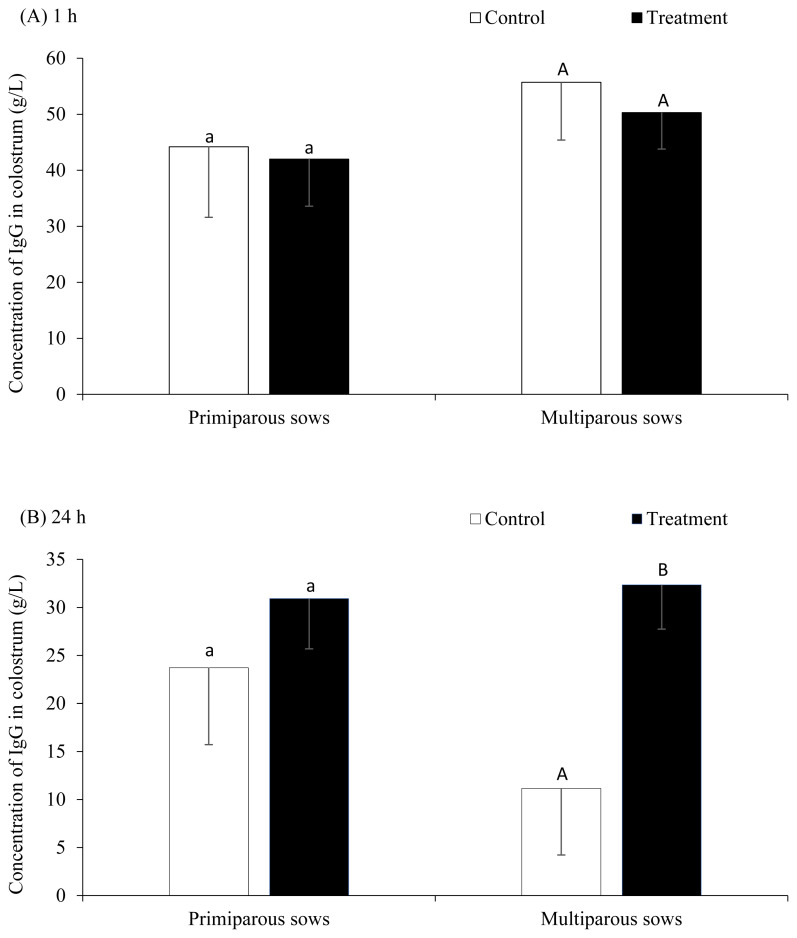
Concentration (g/L) of immunoglobulin G (IgG) in sow colostrum at (A) 1 h and (B) 24 h after farrowing in control and treatment groups by parity groups (n = 26 sows). a,A,B Different superscripts within groups differ significantly (p<0.05).

At 1 h after farrowing, there was no difference of IgG concentration in colostrum between control and PGF2α groups (49.9±8.1 and 46.2±5.3 g/L, respectively, p>0.05) (Figure 1A). However, the IgG concentration at 24 h after farrowing was higher in the PGF2α treated sows compared to the control sows (17.4±5.3 and 31.6±3.5 g/L, respectively, p<0.05) (Figure 1B). Figure 1 demonstrates the concentration of IgG at 1 and 24 h after farrowing in control compared with PGF2α groups in primiparous and multiparous sows. It was found that the difference of IgG concentration at 24 h after farrowing between control and treatment groups was significant in multiparous but not in primiparous sows (Figure 1B). On average, the declining of IgG concentrations from 1 to 24 h after farrowing were 23.5 g/L (i.e., declined 45.4%, p = 0.021) in multiparous sows and 10.7 g/L (i.e., declined 25.1%, p = 0.286) in primiparous sows. Interestingly, in multiparous sows, the IgG concentrations declined significantly during the first 24 h postpartum from 56.7 to 11.2 g/L (i.e., declined 80.2%) in the control group (p = 0.044), but it was not significant in the treatment group (i.e., from 50.3 to 32.4 g/L or declined 17.9%, p = 0.205). Likewise, insignificant tendencies were also observed in primiparous sows (i.e., declined 46.3% and 26.4% in control and treatment groups, respectively, p>0.05). These data indicated that the PGF2α treatment could decelerate the declining of IgG concentrations during the first 24 h postpartum.

### Milk yield

The estimated milk yield of sows is presented in Table 2. On average, the milk yield of sows was 9.28±0.46, 9.84±0.42, and 10.38±0.48 kg/sow/d in control, single PGF2α and multiple PGF2α, respectively (p>0.05). On average, milk yield of primiparous sows was lower than multiparous sows (8.95±0.42 and 10.72±0.32, respectively, p = 0.002). The milk yield of control sows compared with single and multiple treatment with PGF2α by parity are presented in Table 2. For primiparous sows, milk yield was highest in the sows treated with multiple doses of PGF2α during lactation and lowest in control sows (10.25±0.83 and 7.61±0.72 kg, respectively, p = 0.023). For multiparous sows, no difference was observed among groups (Table 2).

### Piglets’ performances

Piglet performances of sows after single and multiple PGF2α treatment compared with control sows are presented in Table 3. On average, litter sizes at days 5 and 20 of lactation were 10.0±1.72 and 9.3±1.46 piglets/L, respectively. Litter weight at 20 days of lactation, litter weight gain, ADG and body weight of the piglets at weaning were 67.2±13.6 kg, 46.7±11.5 kg, 335.8±58.4 g/d, and 7.2±0.92 kg, respectively. The litter weight at day 20 of lactation tended to be higher in the multiple PGF2α group compared with the single PGF2α (p = 0.079) and control sows (p = 0.057) (Table 3). The influence of PGF2α treatment on litter weight at 20 days of lactation was more pronounced in primiparous than multiparous sows. Primiparous sows treated with multiple doses of PGF2α during lactation had a higher litter weight at 20 days of lactation than controls (p = 0.004) and tended to be higher than the single dose of PGF2α (p = 0.075). However, no difference among treatment groups was observed in multiparous sows (p>0.05). The ADG of piglets was highest in multiparous sows treated with multiple doses of PGF2α and lowest in primiparous sows without any treatment (i.e. control) (p = 0.015). Litter weight at 20 days of lactation (p = 0.001), litter weight gain (p = 0.001), ADG (p = 0.050) and body weight of the piglets at weaning (p = 0.066) in multiparous sows was higher than primiparous sows.

### Serum progesterone

On average, serum progesterone of sows during farrowing was 140.6±159.8 nmol/L (mean±SD). The levels of serum progesterone during farrowing varied among sows from 14.2 to 911.6 nmol/L. The levels of serum progesterone were 4.5± 4.7 nmol/L (range 0.16 to 20.5 nmol/L) and 3.0±4.4 nmol/L (range 0.06 to 23.5 nmol/L) at seven and 21 days postpartum, respectively. The data concerning serum progesterone levels by groups are presented in Table 4. There was a significant drop of progesterone after farrowing (p<0.05). The progesterone levels were not different between control and single or multiple PGF2α treated sows (Table 4).

## DISCUSSION

In general, colostrum production in sows is highly variable due to differences in breed, nutrition, sows, number of live-born piglets within the litter, farrowing duration, hormones (i.e. progesterone, prolactin, and oxytocin) and environmental factors [15–19]. The present study demonstrated that the colostrum yield of sows is highly variable and ranges from 0.66 to 9.11 kg. This agrees with earlier studies [1,2,7,8,16,17]. We found that the colostrum yield in multiparous sows was higher than primiparous sows and the treatment with PGF2α postpartum significantly improved colostrum yield in sows. This may be because the treatment with PGF2α postpartum increased uterine contractions and eliminated uterine debris postpartum. Thus, the risk of endometritis is reduced and the potential for colostrogenesis is improved. This is in line with a number of earlier studies which demonstrate that PGF2α treatment in postpartum sows could reduce weaning-to-oestrus interval [20], improve litter size in the subsequent litter [9], increase body weight of piglets at weaning and reduce neonatal mortality [21]. Moreover, the effect of high ambient temperature is of concern regarding colostrum production because heat stress may affect the endocrine status of the sow. The colostrum production of sows is regulated by hormones, including progesterone, prolactin and oxytocin [22]. Heat stress may increase the level of cortical hormones and influence the oxytocin released during parturition, and subsequently increase farrowing duration and reduce colostrum production. Hasan et al [19] found that colostrum production was significantly associated with litter size and the duration of farrowing. Sows with a long duration of farrowing had reduced colostrum production [19]. Hasan et al [19] stated that the sows with too long a duration of farrowing may secrete opioids due to stress and hence, inhibit prolactin secretion and reduce the colostrum production. Moreover, a high concentration of progesterone at the onset of farrowing inhibits the secretion of prolactin [22] and hence, reduces colostrum production [2]. These findings indicate the impact of stress as well as hormonal changes during farrowing on the colostrum production of sows. Additionally, sow colostrum yield is positively correlated with the number of live-born piglets [19]. An additional individual live-born piglet increases the colostrum yield of a sow by 93.6 g [19]. In the present study, the adjusted colostrum yield of sows was calculated by balancing the number of live-born piglets between the control and treatment groups. It was found that after the number of live-born piglets had been balanced, the treatment with PGF2α postpartum still improved the colostrum yield of sows (+1.89 kg). The PGF2α treatment significantly increased colostrum yield by 1.89 kg in multiparous sows, but in primiparous sows, the same treatment insignificantly elevated the colostrum yield by 1.91 kg (from 4.70 to 6.61 kg). Although the same tendency was observed in both primiparous and multiparous sows, the differences in primiparous sows was not significant. The reason could be due to a limited number of observations of primiparous sows. Therefore, further study should be focused on the efficacy of postpartum PGF2α treatment in primiparous sows.

In the present study, the colostrum intake by piglets also varied among individual piglets from 66 up to 987 g per piglet. This is in agreement with previous studies [1,19]. The proportion of piglet preweaning mortality was increased when the piglets received <400 g of colostrum [1]. In the present study, 8.0% of the piglets received inadequate colostrum intake during the first day of life. It is well established that body weight at birth of the piglets strongly influence their ability to consume colostrum [1,23]. In Europe, the average colostrum intake of piglet was 371 g [23], while the average colostrum intake of piglets in the control group in the present study was 563.9 g (Table 2). In the European study, the average number of piglets born alive per litter was 14.0 piglets and the mean body weight at birth of the piglet was 1.28 kg [23], while the same parameters in the present study was 11.1 piglets and 1.34 kg, respectively. The colostrum intake was lower when oxytocin was administered to the sows during farrowing and with increased litter size [23]. The mean colostrum intake of Danbred piglets with a mean birth weight and the sow that was not injected with oxytocin was 570 g with 95% confidence interval (CI) of 472 to 667 g [23]. Therefore, the mean colostrum intake of piglets in the control group in the present study (i.e., 563.9 g) is within 95% CI of the colostrum intake in the European breed. Moreover, colostrum intake decreased on average 9 g for each additional live-born piglet [23] and colostrum intake was also positively associated with birth weight. For instance, in Danbred piglets, colostrum intake increased on average 15.2 g with each increase of 100 g in birth weight [23]. Likewise, in the current study, colostrum intake increased on average 17.5 g (p<0.001) with each increase of 100 g in birth weight. Therefore, the higher colostrum intake of piglets observed in the present study compared to that reported by Declerck et al [23] could be due to a lower litter size and a higher average birth weight of piglets. After adjusting the body weight of piglet at birth in the statistical model (i.e., balanced body weight of piglets between control and treatment groups), the colostrum intake of the control piglets was 563.9 g, lower (56.8 g, p<0.05) than the treatment (620.7 g) indicating significant effect was exerted by the PGF2α injection (Table 2). Earlier studies indicate that the ability of sows to produce adequate colostrum for their piglets during the first day of life influences piglet survival [1,19]. In the current study, administration of PGF2α in sows during the postpartum period improves colostrum production. This might be associated with one or a combination of mechanisms including: i) an increase of smooth muscle contraction of the mammary glands of sows in response to the PGF2α treatment; ii) a reduction of progesterone in some sows by causing luteolysis in the porcine ovaries (see below); and iii.) an excretion of uterine lochia in some sows with a long duration of farrowing.

The present study indicates that the concentration of colostrum IgG declines rapidly within the first 24 h after the onset of farrowing, in agreement with the previous studies [1,24]. This indicates the importance of peri-and post-farrowing management of newborn piglets to enhance their colostrum consumption as soon as possible after birth. Interestingly, administration of PGF2α during the postpartum period could enhance IgG concentration of sows at 24 h after farrowing, even though no difference at 1 h after farrowing was detected. This indicates that administration of PGF2α postpartum can improve the efficacy of colostrogenesis and/or extend the duration of IgG secretion from the mammary glands. This might be associated with the delayed decrease in serum progesterone concentration in sows, which may inhibit prolactin production and therefore compromise the quality of colostrum. Farmer and Quesnel [25] found that the low production of colostrum in sows (i.e. <1.0 kg) was associated with a delayed decrease in progesterone concentration in the blood before the beginning of farrowing. Since PGF2α has a luteolytic action and promotes a rapid decline in progesterone concentration, administration of PGF2α during the postpartum period may help to eliminate excessive corpora lutea in the ovary and reduce progesterone production in postpartum sows. In addition, we demonstrated that the PGF2α treatment could decelerate the declining of IgG concentrations during the first 24 h postpartum. For instance, in multiparous sows, the IgG concentrations declined 80.2% (from 56.7 to 11.2 g/L) from 1 to 24 h postpartum in the control group, but it was declined only 17.9% (from 50.3 to 32.4 g/L) in the treatment group. These data indicated that the declining rate of IgG in the control and treatment groups were 3.5% (i.e., 80.2%/23 h) and 0.8% (i.e., 17.9%/23 h) per hour, respectively. The exponential decay calculation was 7.05% (nature log [LN] [11.2/56.7]/23 h) and 1.91% (LN (32.4/50.3)/23 h) per hour decreasing in the control and treatment groups, respectively. Therefore, the estimated half-life of IgG decline could be 9.8 h (i.e., 0.693/0.0705) and 36.2 h (0.693/0.0191) in the control and treatment groups, respectively. A number of earlier studies indicated that within 10 to 12 h after the onset of parturition, the colostrum IgG levels in sows were reduced by 50% (half-life) and after 24 h a 70% reduction was commonly observed [16,26,27]. However, a high variation of IgG concentrations at 24 h after farrowing are still commonly observed among studies and among sows within the same study and the reason remain unknown. The novelty of the present study was that the PGFα treatment could quantitatively (colostrum yield) and qualitatively (IgG) benefit the sow’s colostrogenesis and copious treatments of PGFα could subsequently stimulate the galactopoiesis.

In the present study, the concentration of serum progesterone during the farrowing process was relatively high (average 140.6 nmol/L or 44.2 ng/mL) compared to previous studies in Finland (3.8 ng/mL) [19] and Canada (8.4 ng/mL). Generally, serum progesterone concentration in sows should have declined sharply to a level of <1.0 ng/mL (i.e., 3.18 nmol/L) within 24 h after farrowing [28]. However, the concentration of progesterone after farrowing varied greatly among sows [29]. The source of progesterone in postpartum sows may come from the remaining corpora lutea, placenta and/or body fat [30]. A previous study demonstrated that progesterone concentration in fat was ten times higher than that in blood [31]. Moreover, piglets suckling sows with a high progesterone level have a reduced body weight gain during the first day postpartum [29]. Similarly, a negative correlation between sow plasma progesterone concentration during the first 48 h postpartum and piglet growth rate during the first three days of life has been demonstrated [29]. These findings indicate that the process of colostrogenesis may be compromised by the delay of progesterone decline after farrowing in sows. Therefore, the use of PGF2α postpartum can induce luteolysis in some animals that had incomplete luteolysis due to inadequate PGF2α. These studies imply that the use of PGF2α treatment within an hour postpartum improves colostrum yield of sows and litter weight gain and possibly decreases piglet mortality by rapidly removing the corpora lutea of pregnancy and eliminating the negative effect of high progesterone on colostrum production. In a previous study, sows kept in a loose house with an abundance of nesting materials and space had a lower serum progesterone at the onset of farrowing than those kept in crates and who had limited access to or no nesting material [19]. In the herd where the present study was conducted, the sows were kept in crates and no nesting materials were used. Therefore, the serum progesterone in sows after the onset of farrowing was relatively high. The high level of progesterone may inhibit prolactin secretion and hence reduce colostrum production. Therefore, administration of PGF2α during the postpartum period may be beneficial for improving the quality and quantity of colostrum.

Across the treatment groups, colostrum yield of multiparous sows was higher than primiparous sows (Table 1). Likewise, milk yield of multiparous sows was also higher than primiparous sows (Table 2). However, in multiparous sows, the PGF2α treatment, either single or multiple, exerted a little effect on milk yield. On the other hand, the PGF2α treatment significantly improved milk yield in primiparous sows (Table 2). This is also indicated by the similarity of average daily gain of piglets and litter weight gain in primiparous sows (Table 3). However, in Table 1, the PGF2α treatment improved colostrum yield in both multiparous sows (from 5.53 to 7.42, i.e., +1.89 kg or 34.2%) and primiparous sows (from 4.70 to 6.61, i.e., +1.91 kg or 40.6%). Although the statistical significance could not be demonstrated in primiparous sows, the numerical improvement was still of interest to perform additional works focusing on the impact of PGF2α treatment in primiparous sows.

In conclusion, postpartum treatment with PGF2α improved colostrum yield and IgG concentration at 24 h in multiparous sows and increased colostrum intake of piglets. Multiple administration of PGF2α at days 0, 7, and 14 postpartum significantly improved the milk yield of primiparous sows and increased the litter weight of piglets at 20 days of lactation.

## Figures and Tables

**Table 1:** Colostrum yield, IgG concentration and piglet performances of sows after prostaglandin F2α treatment compared with control sows (least square means±SEM)

Variables	Control	PGF2α treatment
Number of sows	11	25
Number of piglets born alive per litter	11.8±1.0^a^	12.3±0.4^a^
Piglet birth weight (g)	1,383±36.0^a^	1,331±18.5^a^
Adjusted colostrum yield (kg)^1)^	5.12±0.83^a^	7.01±0.38^b^
Primiparous sows (n = 10)	4.70±1.55^a,A^	6.61±0.63^a,A^
Multiparous sows (n = 16)	5.53±0.59^a,A^	7.42±0.43^b,A^
IgG at 1 h postpartum (g/L)	49.9±8.1^a^	46.2±5.3^a^
Primiparous sows (n = 13)	44.2±12.6^a,A^	42.0±8.4^a,A^
Multiparous sows (n = 21)	56.7±10.3^a,A^	50.3±6.5^a,A^
IgG at 24 h postpartum (g/L)	17.4±5.3^a^	31.6±3.5^b^
Primiparous sows (n = 10)	23.7±8.0^a,A^	30.9±5.2^a,A^
Multiparous sows (n = 13)	11.2±6.9^a,A^	32.4±4.6^b,A^
Adjusted farrowing duration (min)^1)^ (n = 26)	197.1±48.8^a^	199.4±20.2^a^
Backfat thickness (mm)	21.3±0.7^a^	21.0±0.5^a^
Piglet characteristics		
Number of nursed piglets (piglet/litter)	9.5±0.5^a^	9.9±0.4^a^
Birth interval (min)	13.3±2.4^a^	15.6±1.6^a^
Colostrum intake (g)	563.9±20.3^a^	620.7±10.0^b^

IgG, immunoglobulin G; PGF2α, prostaglandin F2α; SEM, standard error of the means.

1) The litters with number of piglets born alive below eight were excluded (n = 6) and sows that used oxytocin were excluded (n = 4).

a,b Different superscripts within rows differ significantly (p<0.05).

A,B Different superscripts within columns differ significantly (p<0.05).

**Table 2:** Milk yield (kg) in primiparous and multiparous sows after single and multiple treatment with PGF2α compared with control sows (least square means±SEM)

Group	Primiparous sows	Multiparous sows
Control	7.61±0.72^a,A^	10.95±0.59^bA^
Single treatment	8.98±0.64^a,AB^	10.69±0.54^b,A^
Multiple treatment	10.25±0.83^a,B^	10.51±0.51^a,A^
All	8.94±0.42^a^	10.72±0.32^b^

PGF2α, prostaglandin F2α; SEM, standard error of the means.

a,b Different superscripts within rows differ significantly (p<0.05).

A,B Different superscripts within columns differ significantly (p<0.05).

**Table 3:** Piglet performance after single and multiple PGF2α treatment in postpartum sows compared with control sows (least square means±SEM)

Variables	Control	PGF2α	

		Single treatment	Multiple treatment
Litter weight at 20 days (kg)	61.9±3.4^a^	63.1±3.1^ab^	71.3±3.3^b^
Primiparous sows (n = 12)	47.1±5.4^aA^	57.5±4.8^abA^	71.0±5.4^bA^
Multiparous sows (n = 23)	76.8±4.1^aB^	68.6±3.8^aA^	71.7±3.8^aA^
Body weight at weaning (kg)	7.2±0.3^ab^	6.8±0.2^a^	7.6±0.2^b^
Primiparous sows (n = 12)	6.5±0.4^aA^	6.8±0.4^aA^	7.5±0.4^aA^
Multiparous sows (n = 23)	7.8±0.3^aB^	6.8±0.3^bA^	7.8±0.3^aA^
Average daily gain (g/d)	322.0±16.9^a^	315.2±16.3^a^	356.4±18.2^a^
Primiparous sows (n = 12)	277.4±26.9^aA^	306.6±24.1^aA^	350.2±31.1^aA^
Multiparous sows (n = 23)	366.6±20.3^aB^	324.0±19.0^aA^	362.6±19.0^aA^
Litter weight gain (kg/d)	42.1±2.9^a^	44.2±2.7^a^	48.9±3.2^a^
Primiparous sows (n = 12)	30.0±4.7^aA^	39.0±4.2^abA^	47.6±5.4^bA^
Multiparous sows (n = 23)	54.3±3.5^aB^	49.3±3.3^aA^	50.2±3.3^aA^

SEM, standard error of the means; PGF2α, prostaglandin F2α.

a,b Different superscripts within rows differ significantly (p<0.05).

A,B Different superscripts within columns differ significantly (p<0.05).

**Table 4:** Serum progesterone (nmol/L) in sows after single and multiple treatment with PGF2α compared with control sows (least square means±SEM)

Days postpartum	Control	PGF2α	

		Single treatment	Multiple treatment
0	183.2±56.7^a^	152.8±14.4^a^	95.4±6.1^a^
7	5.2±53.7^b^	5.9±14.3^b^	2.4±6.1^b^
21	4.3±60.4^b^	3.3±14.9^b^	1.9±6.1^b^

SEM, standard error of the means; PGF2α, prostaglandin F2α.

a,b Different superscripts within columns differ significantly (p<0.05).
